# Glioma Revisited: From Neurogenesis and Cancer Stem Cells to the Epigenetic Regulation of the Niche

**DOI:** 10.1155/2012/537861

**Published:** 2012-07-08

**Authors:** Felipe de Almeida Sassi, Algemir Lunardi Brunetto, Gilberto Schwartsmann, Rafael Roesler, Ana Lucia Abujamra

**Affiliations:** ^1^Cancer Research Laboratory, University Hospital Research Center (CPE-HCPA), Federal University of Rio Grande do Sul, 90035-903 Porto Alegre, RS, Brazil; ^2^Laboratory of Neuropharmacology and Neural Tumor Biology, Department of Pharmacology, Institute for Basic Health Sciences, Federal University of Rio Grande do Sul, 90035-903 Porto Alegre, RS, Brazil; ^3^Children's Cancer Institute and Pediatric Oncology Unit, Federal University Hospital (HCPA), 90035-903 Porto Alegre, RS, Brazil; ^4^National Institute for Translational Medicine (INCT-TM), 90035-903 Porto Alegre, RS, Brazil; ^5^Department of Internal Medicine, School of Medicine, Federal University of Rio Grande do Sul, 90035-903 Porto Alegre, RS, Brazil; ^6^Medical Sciences Program, School of Medicine, Federal University of Rio Grande do Sul, 90035-903 Porto Alegre, RS, Brazil

## Abstract

Gliomas are the most incident brain tumor in adults. This malignancy has very low survival rates, even when combining radio- and chemotherapy. Among the gliomas, glioblastoma multiforme (GBM) is the most common and aggressive type, and patients frequently relapse or become refractory to conventional therapies. The fact that such an aggressive tumor can arise in such a carefully orchestrated organ, where cellular proliferation is barely needed to maintain its function, is a question that has intrigued scientists until very recently, when the discovery of the existence of proliferative cells in the brain overcame such challenges. Even so, the precise origin of gliomas still remains elusive. Thanks to new advents in molecular biology, researchers have been able to depict the first steps of glioma formation and to accumulate knowledge about how neural stem cells and its progenitors become gliomas. Indeed, GBM are composed of a very heterogeneous population of cells, which exhibit a plethora of tumorigenic properties, supporting the presence of cancer stem cells (CSCs) in these tumors. This paper provides a comprehensive analysis of how gliomas initiate and progress, taking into account the role of epigenetic modulation in the crosstalk of cancer cells with their environment.

## 1. Introduction

Gliomas are the most common brain tumor in adults, with very low survival rates, even when combining radio-and chemotherapy. Among the gliomas, glioblastoma multiforme (GBMs) is the most common and aggressive type, and patients frequently relapse or become refractory to conventional therapies. GBMs are usually detected upon the incidence of neurological symptoms, rendering it a disease that is diagnosed already at an advanced stage. Other glioma types include astrocytomas, oligodendrogliomas, and mixed oligoastrocytomas, which are characterized according to their histological features.

How is it that such a malignancy arises in this carefully orchestrated organ, where cellular proliferation is barely needed to maintain its function? This question has intrigued scientists until very recently, when the discovery of the existence of proliferative cells in the brain overcame such doubts. Even so, the precise origin of gliomas still remains elusive. Fortunately, with new advances in molecular biology, researchers have been able to depict the first steps of glioma formation and to accumulate knowledge about how neural stem cells and its progenitors become gliomas. Indeed, GBMs are composed of a very heterogeneous population of cells, which exhibit a plethora of tumorigenic properties, supporting the presence of cancer stem cells (CSCs) in these tumors.

In this paper a comprehensive analysis of how gliomas initiate and progress will be depicted. Several reports have already described each aspect of glioma formation separately. Here, however, we promote an overall landscape of this process, considering how the tumor environment, including its epigenetic mechanisms, may contribute to this disease, providing new insights for better therapeutic approaches.

It is important to understand normal physiological conditions before understanding the pathology itself. Therefore, this paper describes both physiological and pathological conditions together, to better relate the tumor microenvironment studies. There are five parts to this paper, ranging from the historical perspectives of the most relevant works in the field to a discussion of the role of epigenetic modulation in the crosstalk of cancer cells with their environment.

## 2. Part I: Physiological Neurogenic Niches

Before understanding the dynamics of tumor environment and its relationship with cancer cells, it is necessary to depict the physiological conditions in the brain which permit cellular proliferation and stemness, which are necessary for malignant transformation. Many normal stem cell niches from different tissues provide bright information about tumor stem cell behavior, in part because very often tumor stem cells are derived from stem cells of the same tissue of origin and because they may require the same signals to maintain themselves and proliferate in their microenvironment.

Although adult neurogenesis has been extensively discussed over the last century, it was only in 1998 that researchers in the field found *in vivo* evidence for human neurogenesis by screening postmortem brain tissues with the mitotic label bromodeoxyuridine (BrdU) [1], reviewed in [2]. Those findings have pushed brain tumor research to a new level, since it was clear that the brain indeed possessed a source of stem cells, corroborating the thoughts that tumors are most likely originated from cells capable of proliferation (the other possible way being through dedifferentiation, in other words, when a more differentiated cell acquires the phenotype of a stem cell).

Adult neurogenesis is a complex process comprising the activation of a pool of stem cells, the proliferation of precursors, and the differentiation and functional maturity of the newborn cells. Postnatal neuronal production seems to be important not only in pathological conditions, such as epilepsy, ischemia, schizophrenia, and tumorigenesis, but also in normal functions such as learning, memory, and migration (reviewed in [2]). In the adult mammalian brain, neurogenesis is restricted to two areas. The most examined and largest niche is the subventricular zone (SVZ) of the forebrain, followed by the subgranular zone (SGZ) of the hippocampus.

### 2.1. Components of Neurogenic Niches

The SVZ is located in the lateral walls of the lateral ventricles and can be divided into four distinct layers based on its histological structure. The third layer is the most relevant one, since it consists of three distinct astrocyte cell types which participate in neurogenesis: stem cell astrocytes (type B cells, expressing glial fibrillary acidic protein; GFAP+) are more likely to be quiescent; however, they can be stimulated to generate neuroblasts (type A cells, GFAP−/Dlx2+/doublecortin+) through the rapidly dividing transit-amplifying cells (type C cells, GFAP−/Dlx2+) [3]. Neuroblasts originated in the SVZ migrate long distances along the rostral migratory stream (RMS) to the olfactory bulb (OB), where the majority differentiate into granule cells and a small population become periglomerular cells [3, 4]. Beside neurons and astrocytes, oligodendrocytes can also be generated in the adult SVZ [5] (reviewed in [6]), and the role of oligodendrocytes precursors in gliomagenesis will be further discussed in the paper. Type B astrocytes have a characteristic apical process contacting the ventricle and a basal process extending to the underlying blood vessels [7]. In addition, they express neural stem cell markers (such as CD133 and nestin) and are labeled with proliferative markers such as Ki67 and phosphohistone H3 [8]. This subpopulation of slowly dividing neural stem cells (NSCs) can proliferate *in vivo*; moreover they can form neurospheres with multipotential and self-renewal abilities *in vitro* [7, 9], reviewed in [2, 8].

The neurosphere assay is the current gold standard for determining the presence of NSCs [10]. By culturing the cells in serum-free, growth factor-supplemented media in low adherent conditions, stem cells divide continually, forming undifferentiated and multipotent spheres denominated neurospheres, which can be dissociated and replated to expand the culture and select the cells with self-renewal capacity. Neurospheres have been isolated from both the SVZ and SGZ, and they were capable of generating cells with neuronal, oligodendrocyte, and glial markers [11]. The neurosphere assay, which will be discussed in this paper, is also important for evaluating the stemness of brain tumor stem cells (tumorsphere assay) as well.

**Figure 1:**
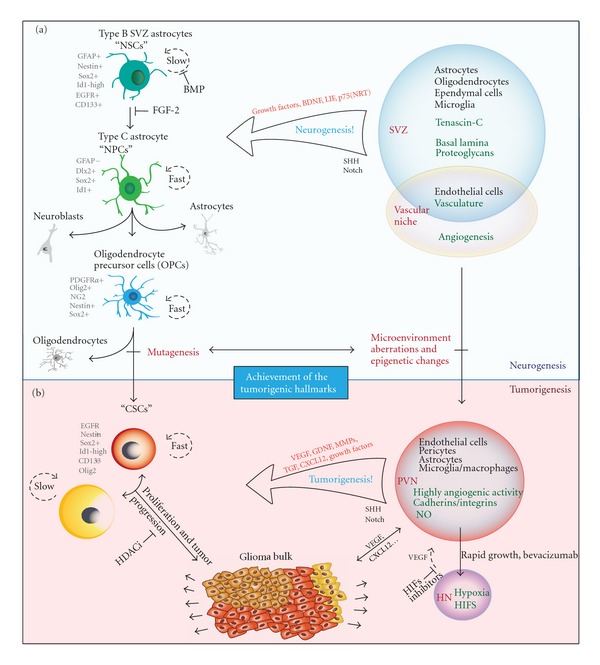
A summary of gliomagenesis. (a) The interactions between the neurogenic niche (subventricular zone, SVZ) and neural stem cells (NSCs) highlighting the most relevant cell types and the secreted factors that affect the neural proliferation. Oligodendrocyte progenitors are more likely to undergo malignant transformation. (b) The role of cancer stem cells (CSCs) in tumor progression. There are different subpopulations of CSCs, which may contribute to tumor heterogeneity. Histone deacetylase inhibitors (HDACis) may be effective against CSCs by promoting their differentiation. The perivascular niches (PVNs) provide growth factors that enhance CSC proliferation and self-renewal. Because of the rapid tumor growth, hypoxic niches (HNs) are formed and, through the action of HIFs, secrete VEGF, which in turn may lead to new vascular niches.

As thoroughly discussed by Quiñones-Hinojosa and collaborators [2, 12], the SVZ is a complex microenvironment composed by different cell types interacting among themselves and with various extracellular molecules that promote neurogenesis. Beside astrocytes, microglia, and oligodendrocytes, endothelial cells also participate in the niche and directly interact with NSCs to enhance neurogenesis *in vitro*. The extracellular matrix (ECM) components, such as tenascin-C, basal lamina components, and chondroitin sulfate proteoglycans [13], also contribute to the neurogenic environment by binding, presenting, and sensitizing various growth and signaling factors to neural precursors (Figure 1(a)).

Furthermore, the cell surface carbohydrate Lewis X (LeX)/CD15 is an epitope that is expressed in all spheres-forming cells from the SVZ and which is shed into their environment, being shown to play an important role in the neurogenic niche modulation by capturing factors from the blood vessels [14].

The hippocampus is another area of the mammalian brain that continues to produce neurons postnatally. Also using BrdU labeling, Kuhn and colleagues [15] confirmed that, in the adult rat brain, neuronal progenitor cells divide at the border between the hilus and the granule cell layer. The newborn granule cells are capable of extending axonal projections along the fiber tract to their natural target area, the hippocampal CA3 region. Two populations of astrocytes have been defined in the SGZ: the radial astrocytes, which extend processes into the granule cell layer, are nestin+ and are able to divide, and the horizontal astrocytes, which extend basal processes under the granule cell layer and are nestin− and S100+ (reviewed in [6]).

In 1998, Eriksson and colleagues [1] finally detected BrdU-labeled cells in the adult human hippocampus, which were quantified in the granule cell layer and the subgranular zone of the dentate gyrus and in the hilus (CA4 region). BrdU-labeled cells also coexpress neuronal markers, indicating the presence of proliferating neural progenitor cells. The newly generated cells were able to survive and differentiate into cells with neuronal morphological and phenotypic characteristics. Cells are generated daily in the young adult rodent dentate gyrus with a fraction integrating into the neuronal circuitry [16]. Current evidence suggests, however, that the proliferating cells of the hippocampus are multipotent progenitor cells instead of NSCs [17, 18].

### 2.2. Neurogenic-Associated Signaling

Most knowledge about the neurogenic niche in the hippocampus came from aging, learning, and memory studies regarding neurogenesis in animal models [14, 15, 18]. Data from numerous studies suggests that precursor activation and neurogenesis are intimately linked to activity levels at the synapse, such as that in the case of voluntary exercise and other novel external sensory experiences [19]. Moreover, long-term potentiation (LTP) has been shown to increase the proliferation of neural precursors in the dentate gyrus [20, 21]. Activation could be through the release of growth factors within the neurogenic niche. Brain-derived neurotrophic factor (BDNF) release is known to be enhanced with electrical activity; therefore it could mediate the synaptic stimulation.

Neurotrophic factors are known to regulate many aspects of the neural cell cycle. BDNF administered to rat SVZ-derived neuroblasts *in vitro* promoted the long-term survival of these cells. Furthermore, following intraventricular infusion of BDNF, other groups also observed increases in the number of newly formed neurons in adjacent structures (reviewed in [22]). In BDNF-null mice, defects in SVZ neurogenesis are not detectable until at least 2 weeks after birth [23]. This indicates that SVZ-derived stem cells destined for the OB may not depend upon BDNF signaling during embryonic and early postnatal development, becoming sensitive to extrinsic factors, such as BDNF, only after birth. Consequently, BDNF is a relevant factor for the survival of adult stem cells and its progeny.

Another neurotrophic receptor participating in adult neurogenesis is the orphan receptor p75(NTR), a member of the tumor necrosis receptor superfamily. p75(NTR) exerts its potent effects on nervous system development through a variety of mechanisms (reviewed in [22]). A high degree of colocalization was found between p75(NTR) and nestin, a marker that labels proliferating cells within the SVZ and RMS. *In vitro* assays show that this population of cells is responsible for the production of all neurospheres and that p75(NTR)-positive cells alone are neurogenic. Beside that, p75(NTR)-null mice show a 70% reduction in their neurogenic potential *in vitro* [22, 24].

It is not a surprise that growth factors play an important role in neuronal proliferation and survival. The fibroblast growth factor (FGF)-2, epidermal growth factor (EGF), transforming growth factor (TGF), ciliary neurotrophic factor (CNTF), and the vascular endothelial growth factor (VEGF) all are able to augment neural proliferation and interfere with neurogenesis (reviewed in [22]). When these growth factors are administrated intraventricularly, they are capable of increasing cellular proliferation, and when their receptors are blocked or knocked down, neurogenesis is significantly affected [25–31].

Platelet-derived growth factor (PDGF) is another key factor, which is known to be a regulator of oligodendrocyte production. PDGFR*α*+ astrocytes are present in the human SVZ [32], and almost 80% of SVZ astrocytes express PDGFR*α* [33]. Studies on the effects of PDGF signaling on neural progenitor cell differentiation demonstrate a proliferating effect on these cells and an inhibition of differentiation [34]. Furthermore, endogenously produced PDGF ligand was detected in cultures, suggesting that this pathway is regulating the proliferation of neural progenitor cells [31]. The vascular-derived molecules also show to locally regulate the adult NSC niche. Some of these molecules include leukemia inhibitory factor (LIF), BDNF, VEGF, and PDGF (reviewed in [35]).

Beside all of these promitotic regulators, studies in rats have elucidated the NSC quiescent mechanism. Researchers have found that quiescent NSCs are induced by autocrine production of bone morphogenetic proteins (BMPs), which induce terminal astrocyte differentiation without EGF and FGF2. Accordingly, the BMP antagonist, noggin, can replace conditioned medium to sustain continuous self-renewal. Noggin can also induce dormant cells to reenter the cell cycle, upon which they reacquire neurogenic potential. The crosstalk between FGF-2 and BMP, which is required to suppress terminal astrocytic differentiation and maintain stem cell potency during dormancy, is crucial to regulate NSCs propagation, dormancy, and differentiation [36] (Figure 1(a)).

Another marker has recently been shown to regulate NSC proliferation. High expression of Id1, a dominant-negative helix-loop-helix transcriptional regulator, identifies a rare population of GFAP+ astrocytes with stem cell attributes among the SVZ. The rare, long-lived, and relatively quiescent Id1^high^ astrocytes self-renew and generate migratory neuroblasts that differentiate into OB interneurons. Cultured Id1^high^ neural stem cells can self-renew asymmetrically and generate a stem and a differentiated cell expressing progressively lower levels of Id1. Id1+ cells, which were also GFAP+ and nestin+, were also evident in the subgranular layer [37].

 Hence, adult neurogenic niches directly rule neuronal production and stem cell maintenance. In contrast, an inhibitory environment that is refractory to neurogenesis is present throughout most of the brain, since primary cells from neurogenic areas transplanted into nonneurogenic regions exhibit very limited neurogenesis (reviewed in [6]).

## 3. Part II: Gliomagenesis

Malignant gliomas, as with any other tumor type, may originate from a complex sequence of events that are necessary to allow the development of these aberrant organs within a normal tissue environment. Multistep tumor formation comprises a cascade that starts with a series of mutations in a pool of susceptible cells and ends with a whole tumor microenvironment that was built during this process and which allows cancer survival and progression. In this context, it is important to comprehend not only the features that a cell acquires to become a cancer cell, but also the role of the microenvironment components, such as different cell types and extrinsic molecules, in the tumor formation process.

### 3.1. First Steps towards Tumorigenesis

Any tumor must achieve basic requirements to successfully develop and grow. Hanahan and Weinberg [38] have logically classified the requirements for tumorigenesis into six basic hallmarks, some of them very remarkable in GBM. First, through the production and release of growth-promoting signals, such as those required for neurogenesis, glioma cells manage to power their own cell cycle and to modulate their own environment in a self-sufficient fashion. Evading growth suppressors comes as an important hallmark as well, since this ability complements the first one: cancer cells must deactivate programs that inhibit cellular proliferation, such as programs that depend on the action of tumor suppressor genes like those which encode for the RB (retinoblastoma-associated) and TP53 proteins. Both proteins act by regulating cellular programs such as proliferation, senescence, and apoptosis. TP53 is also a key regulator of another relevant hallmark, the capacity of resisting cell death signals. In addition, it is widely known that several GBM cell lines present mutant TP53, with variable levels of the protein [39].

In consequence of their rapid growth, tumors demand a larger amount of nutrients and oxygen when compared to normal tissues. These needs are illustrated by the tumor-associated neovasculature, generated by the process of angiogenesis. GBMs are highly angiogenic, and their neoformed vessels are thought to arise from the sprouting of preexisting brain capillaries. Nonetheless, recent findings [40] demonstrate that a population of glioblastoma stem-like cells (GSCs) may originate lineages other than neural lineages. Like normal neural stem cells, which are able to differentiate into functional endothelial cells *in vitro* and *in vivo* [41], *in vitro* cultures of GSCs in endothelial conditions generated progeny with phenotypic and functional features of endothelial cells. The authors have also demonstrated that a significant number of endothelial cells in glioblastoma present the same genomic alteration as tumor cells, indicating that a significant portion of the vascular endothelium has a neoplastic origin. In addition, GSCs closely interact with the vascular niche and promote angiogenesis through the release of VEGF and the chemokine stromal-derived factor 1 (CXCL12) [40]. Therefore, the constituents of signaling cascades and their crosstalks with the tumor microenvironment are crucial for cancer initiation and progression.

Although the necessary components for malignant transformation have been elucidated, the search for the originating cell that leads to glioma formation is still a work in progress. In the past years gliomas were thought to be originated from a transformation of the cell type that is predominant in each tumor, but that remained a speculation. Recently, as adult neurogenesis has been more carefully examined, it was shown that there are still mitotic niches in the postnatal brain. Researchers then focused their efforts on depicting the role of NSCs and neural precursors in glioma formation.

However, studies regarding the first steps towards glioma formation were hampered by the fact that cancer is a dynamic and progressive disease. Consequently, established tumors are just the endpoint of a complex cascade and provide no consistent clues of how they behaved before fully developed. In a very clarifying review [42], Clevers has depicted the role of cancer stem cells (CSCs) in tumor initiation and progression. It was proposed that, as these cells acquire oncogenic mutations, they hierarchically generate subpopulations of cells that have growth advantages among the others, enhancing tumor heterogeneity and dynamics and eventually extinguishing prior subpopulations of cells. Even if the subpopulation derived from the cell of origin persists along the tumor lifespan, its molecular blueprints will be significantly modified as a result of the high genetic instability of cancer cells, hindering its identification. Fortunately, new insights regarding gliomagenesis are emerging, making use of the knowledge acquired from the brain neurogenic niches and the appearance of genetically modified animal models to circumvent the difficulties of working with established tumors. Researchers could, then, for the first time, assess the formation regarding gliomas from the beginning.

### 3.2. The Subventricular Zone as a Tumorigenic Niche

Since the microenvironment in most areas of the brain is repressive to neurogenesis (reviewed in [43]), the neurogenic niches are probably the most vulnerable sites for the growth of transformed cells, since they are abundant in growth factors and thus permissive to proliferation. In addition, they harbor the brain cells with the most proliferative potential, cells that have a higher chance of becoming cancer cells than others [44]. Siebzehnrubl and colleagues [44] propose that the cell of origin of most gliomas may come from the SVZ since this is the largest neurogenic niche, containing the most proliferative cells in the adult brain. Regarding tumor localization, there is evidence that the majority of malignant astrocytic tumors contact the lateral ventricles [45]. The localization of tumors, most of which are benign, away from the lateral ventricles could be explained in part by the existence of progenitor cells away from the niche [46], in contrast with results from another research group which show that more than half of the GBMs studied were radiographically distinct from the ventricles, probably arising from the subcortical white matter and expanding towards the SVZ [47].

Alcantara Llaguno and collaborators [48] used a tamoxifen-inducible nestin−creERT2 transgene to deliver floxed tumor suppressors to the SVZ stem/precursor cells expressing nestin. The results showed that all adult mice subjected to SVZ targeting developed astrocytomas, thus establishing that mutation of these astrocytoma-relevant tumor suppressors in the neurogenic compartment *in vivo* is sufficient to induce tumor formation. They showed that, in contrast to normal adult neural stem cells that are strictly confined to the SVZ or SGZ, tumors arising from these cells or their progeny are not restricted to these niches and indeed migrate away from their normal locations, thus accounting for the presence of tumors elsewhere in the forebrain.

### 3.3. The Search for the Primordial Cell

It is known that stem cells are usually quiescent [49] and proliferate only when demanded. This confers a protective mechanism against transformation, since the more frequently a cell divides, the bigger the chance for it to accumulate mutations and become a cancer cell. On the other hand, progenitors derived from NSCs are a pool of proliferating cells required for neurogenesis. These cells still have the potential to generate different lineages, such as oligodendrocytes and astrocytes, and because they are both multipotent and fast proliferating, they have the highest probability for transformation into a highly malignant tumor [44]. Indeed, type B cells in the SVZ are mainly quiescent and have been found resilient to transformation by c-myc, illustrating that quiescence confers a mechanism of protection [50]. A critical step in neurogenesis which enhances the odds for transformation is the transition of stem cells to transitory amplifying progenitors, a stage involving chromatin rearrangement and a switch from a cell that rarely proliferates to a cell that rapidly proliferates. If genetic lesions are not repaired and persist within these cells, they become incorporated into the dividing population, increasing the chance of further lesions. Whenever a glial progenitor cell reaches the tumorigenic hallmarks, it can result in the dedifferentiation to a more multipotent lineage, such as initiating cancer stem cells, leading to a high-grade glioma. Heterogeneous tumors may also arise from different cell types, because transforming events can affect more than one cell at once. The microenvironment may also increase the probability of transformation in adjacent cells by the release of growth factors [44].

In the same way, gliomas with differing genetic signatures may originate from different cell subtypes [51, 52]. A variety of mutations have been described in human astrocytomas: some of them disrupt cell cycle and apoptosis regulation (INK4A, CDK4, RB, TP53) while others participate in growth factor receptor signaling (EGF, PDGF, PTEN) [52]. More specific genetic models with expression targeted to individual cell types in the SVZ are leading to new insights in brain tumor formation. Such studies exploit particular genetic lesions in the mouse to generate animal models that mimic human malignancy, allowing the investigation of tumor development. Through cre/lox technology, mouse strains with germline or somatic heterozygous mutations at the TP53, NF1, and PTEN tumor suppressor sites developed high-grade astrocytomas with 100% penetrance [53]. TP53, NF1, and PTEN mutations are among the most frequent mutations reported for astrocytomas [50].

In a study by Lee and colleagues [53], human fetal NSCs underwent tumorigenic transformation through the introduction of genes such as v-myc and H-Ras, which resulted in heterogeneous glial tumors with some characteristics of cancer stem cells (small numbers of nestin+ neural stem-like cells). Considering the crucial functions of p53 in protecting cells against oncogenic transformation in a variety of cellular systems, the lower p53 transcriptional activity observed in v-myc-expressing cells may be responsible for the oncogenic transformation induced by the combination of both v-myc and H-Ras genes. Furthermore, this process did not occur when the cells lost neural stemness because of differentiation, indicating that the expression of factors responsible for H-Ras-induced oncogenic transformation may vary according to neural stemness characteristics. This may account for the differing susceptibility to oncogenic transformation between differentiated glial cells and NSCs.

### 3.4. Glial Progenitors as a Plausible Cell of Origin

Although many researchers have successfully focused their studies towards depicting the role of NSCs in gliomagenesis, a remarkable effort has been made along the same lines as those proposed by Siebzehnrubl and colleagues, highlighting the glial progenitor population as being much more susceptible to neoplastic transformation. Some relevant results were pointed by Canoll and Goldman in their review [46], such as the *in vivo* evidence that adult glial progenitors have the proliferative and self-renewing capacity needed to form malignant tumors. These results were obtained by studies that made use of infecting progenitors in the adult white matter with retroviruses that express PDGF, generating tumors that closely resembled human glioblastoma and that were composed of cells bearing the immunophenotype of oligodendrocyte progenitors (olig2+/NG2+/PDGFR*α*+). They also emphasized that glial precursors can be found throughout the brain and can behave in a malignant manner when overstimulated with high levels of growth factors such as PDGF and EGF. Such findings also point out the possibility that cancer stem cells can arise from glial progenitors beside the NSCs with SVZ origins.

Perhaps the most elucidating study regarding the cellular origins of gliomas emerged in 2011 by Liu and colleagues [54] (commented in [55]). Through mosaic analysis with double markers (MADM), they generated a mouse genetic mosaic system to analyze aberrations in individual cell lineages before the final transformation, allowing for the screening of the cell of origin. When mutations are introduced in stem/progenitor cells, it is extremely difficult to distinguish whether initial mutant cells directly transform or whether they simply pass on mutations to more restricted progeny that can undergo further malignant transformation and dedifferentiation into a cancer stem cell. After initiating p53/NF1 mutations sporadically in NSCs, they analyzed mutant NSCs and all of their progeny at pre-malignant stages. The MADM technique allowed Liu et al. to discriminate between cells and its progeny with oncogenic mutations by utilizing a GFP tracer from normal counterparts utilizing a RFP tracer over time. Only mutant NSCs generated neoplastic oligodendrocyte precursor cells (OPCs) which were PDGFR*α*+. All other NSCs-derived cell types, including NSCs themselves, remained mostly unaffected by the disruption of the two tumor suppressive pathways. When p53/Nf1 inactivation is targeted specifically to OPCs, tumors form as NSCs-derived gliomas. Interestingly, these tumors acquired the expression of NSCs genes, which could be misleading during analysis in further stages of the tumor development. The findings demonstrate that, in p53/Nf1 mutation-driven gliomas, mutations may initially occur in either NSCs or OPCs, but only OPCs provide the suitable cellular context needed for transformation.

Their studies emphasize the importance of the intersection between genetic mutations and the signaling context within the cell of origin. Furthermore, they showed that OPCs are particularly sensitive to p53/NF1 mutations, whereas NSCs and other brain cell types are much less responsive, opposing the results obtained by the genetically modified animal model in Lee's studies [53]. Liu's findings have also pointed that nestin−driven mutagenesis results in OPC transformation away from the SVZ, where NSCs reside, with lesions starting at the gray matter and later migrating to the SVZ and white matter, where the tumor fully developed, perhaps with the benefit of the neurogenic niche. It is interesting to see malignant gliomas arising from the gray matter and moving further out, since Canoll's groups have previously shown that the transformation of glial progenitors by PDGF can also result in malignant gliomas in the white matter [56].

## 4. Part III: The Brain Tumor Microenvironment

Cells are continually receiving information from their microenvironment concerning how they should behave, and in the same way cancer cells cannot survive alienated from the surrounding tissues [38]. Once cancer cells start to propagate in their cradle and are established as developed tumors, they manage to construct a complex network in their own microenvironment. In the same way stromal cells from normal tissue restrict the tumor's malignant behavior, cancer cells collaborate for its survival. These feedbacks from both parts are determining for tumor progression or regression. Therefore, there is a constant communication and an intimate relationship between the tumor niche and its surroundings. This network consists of different cellular types beside the cancer cells themselves, being of note the extracellular matrix proteins and soluble signaling factors and cytokines.

Among the various cells present in the tumor bulk, the majority of nontransformed cells in gliomas are tumor-associated macrophages (TAMs, from nonneuronal tissues) and microglia. Macrophages are the predominant inflammatory cells infiltrating gliomas and most of the time microglia stand in the tumor bulk periphery [57]. Evidence suggests that the immune function of microglia might be suppressed when these cells are located inside the tumor, as a result of inflammatory cytokine production, such as interleukin-10 (IL-10), IL-4, IL-6, TGF-*β*, and prostaglandin E2 by cancer cells (reviewed in [58]). TGF-*β* in particular suppresses the activation and proliferation of microglia. Beside that, there is also an impact of microglia on glioma migration which might be related to the production of membrane type 1 metalloproteinase. Microglia in the glioma microenvironment are also a primary source of interleukin 1b (IL-1b), which can enhance gene expression of TGF-*β* [59]. Increased transcription of TGF-*β* can, thus, lead to suppression of antiglioma responses by inhibiting the immune response and blocking antitumor activity [60]. TGF-*β* can also lead to angiogenesis (by enhancing VEGF expression), proliferation (by enhancing EGFR expression), and invasion (by stimulating MMP-9 production) [61]. Therefore, these reports show that when microglia are in a glioma context, they acquire a phenotype that can support tumor development and progression.

The vast metabolic and nutritional needs of gliomas are supplied by constant angiogenic activity, which makes these tumors highly vascularized. The formation of new vessels is a result of the secretion of VEGF by the tumor cells directly and by fibroblasts and inflammatory cells in the stroma. Macrophages also release a number of factors that influence endothelial cell behavior, including VEGF, hepatocyte growth factor (HGF), MMP-2, and cytokines and interleukins [62]. The rapid neovascularization, typical of cancers, often leads to the production of abnormal and non-uniform vessels which eventually produce hypoxic niches that stimulate additional VEGF production [63]. Endothelial cells thus have a relevant role in the progression of brain tumors. Beside promotion and regulation of angiogenesis, they also release factors that maintain the stemness of CSCs, a topic that will be better examined further on in this paper.

Next to the vascular endothelium, there are nontransformed astrocytes, which exert a trophic role in the tumor microenvironment. They secrete a number of neurotrophic factors, including transforming growth factor (TGF-*α*), CXCL12, and glioma-derived growth factor (GDNF). These neurotrophic factors have been described as capable of driving the invasive properties of GBM cells and other aspects of tumor progression, such as angiogenesis, metastasis, and survival of other cancer types [64–66]. Astrocytes are widely recognized components of the blood-brain barrier (BBB), conferring barrier tight junctions with brain endothelial cells. Immunostaining experiments of the astrocyte-endothelial interface of the BBB suggest that tumors induce specific changes in endothelial cells. Abnormal astrocyte-endothelial interactions lead to the remodeling of the ECM, which can thus facilitate tumor invasion [67].

Another endogenous nontransformed cell type that interacts with gliomas is the neural precursor cell (NPC). These cells were demonstrated to migrate towards primary brain tumors over large distances, even when there are only few cancer cells (reviewed in [58]). Large numbers of NPCs were derived from the SVZ and home into pathologic brain tissue and possibly to tumors as well because of CXCR4 expression (the receptor for CXCL12) [68]. Several labeling techniques were used to track endogenous NPCs and identify their presence near gliomas. The genetically labeled cells were accumulated in many cellular layers around gliomas, and further experiments indicated that the precursors were exerting antitumorigenic actions, diminishing glioma proliferation, and leading to glioma cell apoptosis [58, 68, 69]. Chirasani et al. identified the bone morphogenetic protein-7 (BMP7) as an NPC-derived paracrine tumor suppressor that induces the differentiation of human GSCs [70]. Regarding these properties of NPCs, researchers began to explore manipulated NPCs to delivery cytokines, enzymes, and viral particles specifically to cancer cells [71].

Finally, the neural ECM consists of a unique microenvironment within the CNS, with specific molecules and structure. As it is known, the first difference is the absence of fibroblasts and collagen nearly throughout the brain. In turn, the brain ECM is composed mainly of hyaluronan, proteoglycans, tenascin-C, and thrombospondin, which confer a high state of hydration and loose connections (reviewed in [72]). The composition of the ECM in brain tumors is significantly altered. Within primary brain tumors, components such as vitronectin, osteopontin, tenascin-C, SPARC and BEHAB can be found, and some of them are upregulated and modulate brain tumor growth, proliferation and invasion (reviewed in [72]).

## 5. Part IV: Glioma Stem Cells and Their Niches

### 5.1. Glioma Stem Cells Properties and Signaling

Behind tumor initiation, establishment, and dynamic evolution, there is a group of cells that plays a central role in all of these stages: glioma stem cells (GSCs). These cells have been isolated and characterized as a heterogeneous cell population that have unique features, making them a relevant key in tumor survival. They also show marked capacity for proliferation, self-renewal, and differentiation [73]. Characteristic GSCs can be defined according to their ability to efficiently reconstitute the original tumor when transplanted into immunocompromised mice (xenograft assay) [42]. Furthermore, they should express markers that are also expressed by the normal stem cells in the tissue of origin.

CD133 (prominin-1) is a transmembrane glycoprotein that is normally expressed in hematopoietic stem cells, endothelial precursor cells, and NSCs [74–76]. The CD133+ subpopulation of GSCs was demonstrated to present a more malignant behavior: the frequency of CD133+ cells was shown to increase with tumor grade, and its frequency is related to tumor recurrence [77]. Moreover, radioresistant tumors displayed higher percentage of CD133+ cells than the parent cell population, since GSCs could repair the damages more rapidly and efficiently than matched nonstem cells. Therefore, these data demonstrate that CD133+ cells may play an important role in GSC resistance to chemo- and radiotherapy [78]. CD133 is also informative for GSC division mode: in the research conducted by Lathia et al. [79], CD133 was the only marker among others (such as Bmi-1, nestin, CD15, Sox2, and Olig2) that could be asymmetrically segregated, as a result of localized CD133 expression and its positioning against the mitotic axis. The symmetric expansion mode will increase the GSC pool in the tumor, whereas asymmetric cell division will increase cellular heterogeneity of the tumor while maintaining the GSC pool. Other stem cell markers were not cosegregated with CD133. Their study also demonstrated that, in CD133− cells, CD15 could serve as a GSC marker, since this population survive better and proliferate faster as compared to their negative counterparts, complementing some part of CD133 function.

Intrinsic regulation of GSCs occurs through key proliferative and survival pathways including c-Myc, Oct4 (POU5F1), Olig2, and Bmi1, which are known to regulate embryonic stem cell proliferation as well [80]. In the same way notch, sonic hedgehog (SHH), and Wnt are important for the proliferation and stemness of NSCs, as well as for other cancer cells (Figure 1(b)). In Kondo's review [81], three pathways were depicted: Notch receptors are involved in a number of biological functions, including cell proliferation, differentiation, survival, and tumorigenesis [82]. There is also accumulating evidence that Notch activation not only maintains the multipotentiality of NSCs, but also promotes their differentiation into astrocytes. Regarding tumors, the depletion of Notch1 by RNAi blocks glioma proliferation *in vivo* and *in vitro* [83], suggesting that Notch signaling is involved in gliomagenesis, as well as in normal brain development.

SHH signaling is also involved in proliferation, development, and tumorigenesis [84]. Proteins that participate in the SHH pathway, such as Gli, Ptc1, and Smo, are all expressed in the SVZ, suggesting that SHH signaling may be essential for the maintenance of NSCs. Ectopic activation of Hedgehog in the central nervous system is likely to lead to brain tumor formation, and Gli1 is highly activated in many brain cancers [84, 85] (reviewed in [81]). Mutations in the SHH pathway are associated with medulloblastomas, which are primary brain tumors common in children. Hedgehog signaling is active in gliomas and contributes to GSCs function (reviewed in [80]), and its ligands are required for GSCs self-renewal as well as tumorigenesis. Treatment of GSCs with the Hedgehog inhibitor cyclopamine inhibits proliferation and self-renewal while increasing apoptosis [86]. Furthermore, CD133+ glioma cells overexpress genes involved in Notch and SHH pathways. These pathways contribute to the chemoresistant phenotype of CD133+ glioma cells, as their antagonism leads to an additive effect when used in combination with temozolomide (TMZ), which is an oral alkylating antineoplastic agent used for the treatment of GBM [87]. The authors showed that the therapeutic effect of TMZ was enhanced by inhibiting the Notch and SHH pathways with the antagonists GSI-1 and cyclopamine. More importantly, simultaneous treatment involving TMZ with both of these compounds led to a significant increase in CD133+ glioma cytotoxicity when compared to treatment with any of these agents alone.

The Wnt family coordinates several developmental processes, including cell proliferation and cell fate, via secreted proteins [88] (reviewed in [81]). Wnt1 and 3a, for example, are expressed in the ventricular and SVZ in the developing brain. Furthermore, the Wnt-*β*-catenin pathway is also involved in NSCs proliferation [89], and its disregulation has been implicated in many medulloblastomas [90] (reviewed in [2]). These findings suggest that hyperactivation of Wnt signaling may promote brain tumourigenesis.

Extrinsically, GSCs are regulated by growth factors as well as cell-cell and cell-extracellular matrix (ECM) interactions. GSC behavior is constantly affected by external signals from the niche, including neighboring stromal, immune, and nonstem tumor cells. Such signals will trigger the intrinsic pathways above described and will thus regulate CSCs function and properties. Some of these extrinsic pathways are well described: the signal transducer and activator of transcription 3 (STAT3), a member of the STAT family of transcription factors, is important in GBM, tumorigenesis, central nervous system development, and embryonic stem cell (ESC) biology. STAT3 is activated by a wide variety of cytokines and growth factors. STAT3 target genes regulate many cellular processes, including proliferation and apoptosis, and constitutive activation of STAT3 has been observed in many human cancers [91, 92]. Sherry and collaborators [93] have found that treatment of GSCs with two small molecules which prevent DNA binding of STAT3 inhibits, cell proliferation and the formation of new tumorspheres from single cells. Genetic knockdown of STAT3 using a short hairpin RNA also inhibits GSCs proliferation and tumorsphere formation. Markers of neural stem cell, such as Olig2 and nestin, also decrease upon STAT3 inhibition, suggesting that STAT3 is required for maintenance of the stem-like characteristics of these cells.

The RTK (receptor tyrosine kinase) family of receptors mediates the effects of multiple oncogenic growth factor pathways, among which the EGFR is one of the best characterized in gliomas. The signal initiated by RTKs activates the Akt pathway, which promotes survival, proliferation, invasion, and secretion of proangiogenic factors. Pharmacologic inhibitors of Akt attenuate GSC tumorsphere formation, induce apoptosis, and substantially delay intracranial tumor formation [80]. Eyler and collaborators [94] have demonstrated that GSCs are more dependent on Akt signals than matched nonstem glioma cells. Treatment with an Akt inhibitor more potently reduced the numbers of viable brain cancer stem cells relative to matched nonstem cancer cells associated with a preferential induction of apoptosis and a suppression of neurosphere formation. Akt inhibition also reduced the motility and invasiveness of all tumor cells, but had a greater impact on cancer stem cell behavior.

Bone morphogenetic proteins (BMPs) is another family of growth factors that are crucial to regulate differentiation, proliferation, and apoptosis of NSCs [95]. Findings by Sun et al. highlight an extrinsic regulatory network, comprising BMPs, BMP antagonists, and FGF-2 signals, which govern proliferation, dormancy, and differentiation of rat NSCs and which can be manipulated to enable long-term clonogenic self-renewal. BMP induces NSC growth arrest through the canonical effectors Smads, but, in the presence of FGF-2, terminal differentiation is blocked and stem cell potency preserved. These findings indicate that NSC propagation, dormancy, and differentiation are regulated by counterbalancing BMP and FGF signals [36]. The same regulatory network should also be important for GSCs. Indeed, treating GSCs with BMPs *in vivo* markedly delays tumor growth and reduces tumor invasion. These data suggest that selective activation of BMP pathways may reduce the tumorigenic capacity of GSCs [96].

### 5.2. CSCs Contribute to Glioma Cellular Heterogeneity

Remarkably, GBM consists of morphologically diverse cells expressing a wide variety of differentiated and undifferentiated markers [42, 52]. Models that explain the origin of tumor heterogeneity and their capacity to undergo fast malignant progression can be adapted to GBM: the first one consists of a stochastic model in which all tumor cells have a random probability of developing mutations to permit tumor maintenance, and the second is based on a hierarchical model in which sustained tumor growth is restricted to selected subpopulations, such as CSCs [97]. Studies on acute myeloid leukemia have brought useful knowledge concerning the CSC model that could be applied to other tumor types: it is suggested that the tumor is originated from leukemic stem cells that, regarding their self-renewal capacity, are superior in a hierarchical manner to its subsequent progenitors, which are locally restricted to the stem cell niche [42, 98]. However, these models are not mutually exclusive: a single tumor may contain multiple CSC clones that are genetically distinct as a result of the stochastic model, but these cells will always have a common ancestor, the cell that sustained the first oncogenic mutation.

**Figure 2:**
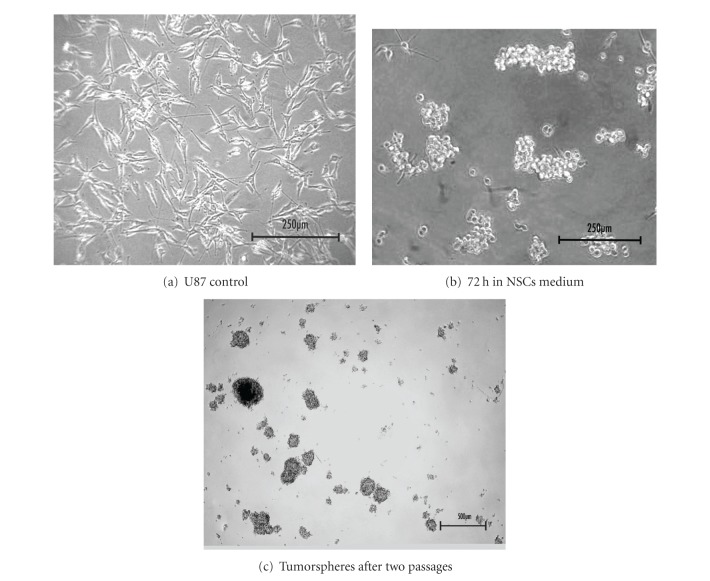
Glioma tumorspheres. (a) U87 glioma cell line in DMEM + 10% of FBS. (b) Tumorspheres of U87 cells after 3 days in NSCs medium. (c) Tumorspheres after two dissociations.

As Clevers has pointed out [42], in order for a certain cancer type to fit into the CSC model, it has to be demonstrated that the primary tumor has different capacities for tumor initiation among the tumor cell subsets, therefore illustrating the presence of CSCs. Singh and collaborators have reported that in human brain tumors there is a cluster of CD133+ cells that could initiate new brain tumors in immunodeficient mice, while CD133− cells could not [73]. Like normal NSCs, GSCs can form spheres when cultured in serum-free medium supplemented with EGF and FGF and could be induced to differentiate into all neuronal lineages expressing mature neuron markers, astrocytes, and oligodendrocytes (reviewed in [99]).  Figure 2 illustrates the resulting tumorspheres, obtained in our laboratory by switching the usual medium to the NSC medium which allows enrichment of stem cell and pluripotency markers through cell growth in suspension [100–102], without the use of cell sorting, which only selects specific GSC subpopulations.

In spite of the recent controversy concerning the use of self-renewal as a tumorigenic marker [103] and the difficulties that are intrinsic to this methodology (such as culture artifacts) [98], the use of the tumorsphere assay to select for GSCs is still widely accepted. However, in order to obtain a more reliable understanding of GSCs behavior, *in vivo* studies concerning the GSC tumor niche should be considered. With that in mind, GSC niches are going to be depicted next.

### 5.3. The Perivascular Niche (PVN)

Since most CSCs usually inhabit a microenvironment very similar to the ones of normal stem cells, we are encouraged to explore both niches to develop new approaches to cancer treatments which specifically target CSCs and their communication with the microenvironment. In GBM GSCs have been localized in two distinct niches, which are going to be discussed in this part of the paper.

The study of neurogenic niches in mammalians has led to the first thoughts regarding the existence of a particular niche in brain tumors in which CSCs could reside. These studies provided solid reports about the importance of the vasculature for neurogenesis: the vascular compartment within the neural stem cell niche was shown to have the unique capacity to regulate neural stem and progenitor cells through direct contact and paracrine signaling by endothelial and mural cells, also integrating systemic signals into the local microenvironment via distribution of soluble factors in the circulation to regulate stem cell niche behavior (reviewed in [35]). These thoughts, together with the fact that the most aggressive brain tumors present an overwhelming angiogenic activity (endothelial hyperplasia and microvascular proliferation) [104], have led scientists to investigate in more detail the location of GSCs within the tumor, making use of NSC markers such as those discussed above.

In 2007, Calabrese and colleagues [105] published an elegant report elucidating the role of the vasculature in brain tumor stem cells (BTSCs). Their data support the hypothesis that vascular niches in brain tumors are abnormal and contribute directly to the generation of GSCs and tumor growth (Figure 1(b)). They have found that many of the vessel-associated nestin+ tumor cells are proliferating and interacting with endothelial cells and that endothelial cells maintain self-renewal of BTSCs in culture and promote the initiation and growth of orthotopic brain tumor xenografts (with GFP-labeled CD133+ cells). Endothelial cocultures also demonstrated that endothelial cells maintain self-renewing and undifferentiated BTSCs. In addition, several molecular signaling events from endothelial cells and other stromal cells within the perivascular microenvironment appear to regulate the stem cell-like properties of resident BTSCs, in a very similar way as that seen in the NSCs niches (reviewed in [58]).

Very recently, human glioma tissue samples were analyzed by immunohistochemistry assays by He and collaborators [106]. They noted that CD133+ and nestin+ niches are localized perivascularly in all glioma tissues and that blood vessels were also nestin− and CD133+. Both CD133+ blood vessels and nestin+ blood vessels have an important role in maintaining glioma stem cell niche structure. Moreover, the abundance of CD133+ niches and nestin+ niches increases significantly as tumor grade increases.

It is important to point out that the relationship between GSCs and their microenvironment is reciprocal: GSCs are able to modulate the same microenvironment that produces the signals that regulate themselves. For example, GSCs secrete VEGF, which stimulates endothelial cell growth to support a local vascular environment. In turn, endothelial cells express Notch ligands which stimulate Notch receptors, which are essential for GSCs maintenance (reviewed in [107]). GSCs have a stronger capacity for promoting angiogenesis, partially through amplified secretion of VEGF, compared to noncancer stem cells [108]. Treating GSCs with the VEGF-neutralizing antibody bevacizumab attenuates their ability to promote angiogenesis both *in vitro* and *in vivo*, which in turn markedly inhibits the GSC tumorigenesis (reviewed in [83]).

The perivascular niche has also been shown to regulate GSC phenotype through regulation of the Notch pathway in these cells (reviewed in [76]). Blockade of this pathway has been demonstrated to deplete GSC population through reduced proliferation and increased apoptosis, as well as through increase in the sensitivity of GSCs to radiation-induced cell death, underscoring the importance of Notch in the regulation of GSCs. Nitric oxide (NO) is another factor in the PVN with the capacity to enhance the self-renewal characteristics of BTSCs. NO also activates Notch signaling in the BTSCs to enhance their self-renewal characteristics *in vitro* and their tumorigenic capacities *in vivo*. Further, eNOS, an enzyme that synthesizes NO from the vascular endothelium, is elevated in the platelet-derived growth factor (PDGF)+ subset of gliomas, and suppression of eNOS activity, which corresponded to a decrease in Notch signaling in these tumors, prolonged survival of tumor-bearing mice [109] (reviewed in [58]).

Surrounding the vasculature are large-body, GFAP-expressing astrocytes and smooth muscle actin-expressing fibroblastic pericytes that intimately associate with tumor endothelia. Macrophages are also located in this region and are recognized to play a significant role in tumor progression of many tumor types [58]. In the normal neurogenic microenvironments, rates of cell proliferation are quite low. On the other hand, researchers found that many of BTSCs in niches were proliferating, differentiating, and benefiting from the protection of their niche through the adherence of stem cells to the niche by cadherin- and integrin-mediated cell adhesion, molecules which are enriched in GSCs [82, 105, 106].

It is thus clear that the tumor microvasculature generates specific niche microenvironments that promote the establishment and maintenance of BTSCs. As well as regulating stem cell proliferation and cell-fate decisions, niches also play a protective role, shielding stem cells from environmental insults, like chemo- and radiotherapy [81, 105]. The dynamics of the PVN structure have only recently been elucidated however. Beside the usual evidences of angiogenesis and vasculogenesis (tumor vasculature arising from sprouting and proliferation of endothelial cells from local vessels and colonization of circulating endothelial or other cells primarily from the bone marrow, resp.) [110], the reports from Ricci-Vitiani et al. and Wang et al. [40, 111] show that beside the already known features of GSCs, they are also capable of transdifferentiating into endothelial cells. Wang's group has demonstrated that a subpopulation of endothelial cells within glioblastomas harbor the same somatic mutations identified within tumor cells, such as amplification of EGFR and chromosome 7. The stem-cell-like CD133+ fraction includes a subset of vascular endothelial-cadherin (CD144+) cells that show characteristics of endothelial progenitors capable of maturing into endothelial cells [111].

The Ricci-Vitiani group has also demonstrated that a variable number (range 20–90%) of endothelial cells in glioblastoma carry the same genomic alterations as tumor cells, indicating that a significant portion of the vascular endothelium has a neoplastic origin. The vascular endothelium contained a subset of tumorigenic cells that produced highly vascularized anaplastic tumors [40].

In 2011, Lathia and collaborators [112], in a very elegant study, have provided the first direct evidence for tumor propagation by a solid GSC tumor subpopulation *in vivo*. Making use of live imaging, they showed that a small fraction of tumor cells that resided perivascularly initiated a heterogeneous tumor. Through xenotransplantation models, they were able to evaluate the GSC behavior in a niche context, avoiding culture artifacts and considering the niche interactions with components such as the vasculature and stroma. They investigated the behavior of GSCs and nonstem tumor cells in an identical microenvironment, transplanting differentially labeled human GSCs and nonstem tumor cells derived from the same parental tumor into the same recipient mouse and monitored their *in vivo* behavior over time using intravital microscopy. The results showed that GSCs (10% of the total number of transplanted cells) outgrew the nonstem cells population. Intriguingly, the resulting tumors had an overwhelming majority of cells that were derived from GSCs. Furthermore, GSCs and their descendants (YFP-labeled cells) were in proximity to the vasculature. Analysis of Sox2 expression, a GSC marker, showed that 25.9% of transplanted GSCs and their descendants were Sox2+ as compared to 0.1% of nonstem tumor cells and their descendants. Hence it was determined that the transplanted tumor cells contained stem-like cells with capacity to self-renew. Their results also suggest that the *in vivo* environment provides instructive cues to recreate an equilibrium of differentiation status and thus cellular heterogeneity.

### 5.4. The Hypoxic Niche (HN)

Hypoxic niches spontaneously arise in malignant tumors as a result of the fast tumor growth that exceeds its neovascularization [113]. Furthermore, with increasing tumor size, tumor perfusion declines because of the severe morphological and functional alterations of the tumor microcirculation [114] (reviewed in [115]). Whenever the vasculature inefficiently irrigates a tissue, the resultant reduction in tissue oxygen tension often leads to neovascularization to satisfy the tissue's needs [116]. VEGF mRNA levels are increased after exposing different cell cultures to hypoxia, but return to background levels when the normal oxygen supply is resumed. VEGF was then identified as the main factor that mediates this feedback response, functioning as a hypoxia-inducible angiogenic factor [117].

In 1993 researchers were unraveling the cellular response to hypoxia in cancer cells [118]. They found that transcription of the human erythropoietin (EPO) gene is activated in Hep3B cells exposed to hypoxia and that the hypoxia-inducible factor 1 (HIF-1) was the nuclear factor whose DNA binding activity was induced in such conditions (hypoxia prevents proteasomal degradation of cytosolic HIFs). Therefore, they were the molecular mediators of hypoxia. About ten years later, by the time that scientists found CSCs in brain tumors, there was a solid concern about how oxygen levels influence tumor behavior. What they did not know was that the recently discovered subpopulation with stem cell characteristics within the tumor would be ruling this behavior. What they did not know was that the recently discovered subpopulation with stem cell characteristics within the tumor would be ruling this behavior; at the time, it was observed that hypoxia was associated with tumor aggression [119]. Some of the mechanisms they thought to be underlying the relation between hypoxia and tumor aggression were the hypoxic regulation of cytokine and growth factor release, such as VEGF, the regulation of tumor suppressors and oncogenes, and the modulation of invasion-associated cytokines, such as MMP [118].

Rankin and Giaccia [120] have recently reviewed the role of hypoxia in tumorigenesis, given that the expression of both HIF-1*α* and HIF-2*α* are commonly increased in a variety of human tumors. Their study pointed out that HIFs can promote tumorigenesis by the regulation of several hallmarks, such as angiogenesis, metabolism, proliferation, metastasis, and differentiation. The last one is relevant, since HIF indirectly regulates proliferation and differentiation through interactions with other signaling proteins such as c-Myc and Notch, both important for the CSC maintenance. In addition, it is known that normal stem cells reside in regions of low oxygen pressure, such as the hypoxic niche (HN) in the bone marrow, where hematopoietic stem cells proliferate [121].

A very intriguing research by Heddleston and collaborators [122] shows that hypoxia induces the expression of key stem cell genes, specifically Nanog, Oct4, and c-Myc, in nonstem cancer cells (the same genes Yamanaka used to reprogram fibroblasts to induce pluripotent stem cells [123]). Furthermore, they showed that inducing HIF-2*α* expression alone can reprogram differentiated, nonstem cancer cells towards an undifferentiated state, similar to neurospheres, since HIF-2*α* may directly regulate core stem cell pathways that are essential in CSC maintenance.

Another clarifying work by Seidel and colleagues [124] specifically explored the relationship between GSCs and hypoxia. In this research, the authors have isolated and characterized GSCs using a side population assay, defining a differential signature that made it possible to track cells through immunohistochemistry. Signature gene expressions, such as CD133, were located in perinecrotic (hypoxic) areas and in perivascular niches as well. HIF-2*α* overexpression, instead of HIF-1*α*, resulted in a significative increase in the levels of all side population markers tested, as well as of the established HIF-2*α* target, Oct4. HIF-2*α* knockdown in a primary GBM cell line completely blocked the upregulation of the side population signature genes following hypoxia, demonstrating how hypoxia controls the expression of several genes that regulate stem cells.

Regarding the actual therapeutics concerning both niches, the highlights are laid on the humanized monoclonal antibody against vascular endothelial growth factor (VEGF)-A, bevacizumab, which was the first antiangiogenic agent to be approved for cancer therapy in patients with metastatic colorectal cancer, nonsquamous non-small-cell lung cancer, and metastatic breast cancer (reviewed in [125]).

Since glioblastoma are highly vascularized cancers and have high expression of VEGF, bevacizumab seemed a proper choice for treatment. It was shown to improve patient outcomes in combination with chemotherapy in recurrent GBM in two distinct prospective phase 2 studies, granting approval by the US Food and Drug Administration (FDA) as a single agent in recurrent GBM (reviewed in [126]). However, bevacizumab used for metastatic breast cancer has not been shown to provide a benefit for delay in tumor growth and in improving overall survival, forcing the FDA to revoke the agency's accelerated bevacizumab approval for HER2-negative breast cancer. Bevacizumab, however, remained on the market, since it has been approved for the treatment of other cancer types [127].

It is important to point out that the tumor response against an antiangiogenic agent may differ between tumor types and subtypes, and, as a result, the complex mechanisms involved in antiangiogenic therapy are still being uncovered. Treatment of glioblastoma *in vivo* with an antibody against VEGFR-2 has inhibited angiogenesis but has also increased tumor invasiveness along host microvasculature [128]. Since high-grade gliomas often show a remarkable brain invasion capacity, this finding has emphasized the need of a combination of different treatment regimens against glioblastoma. To illustrate, two studies have successfully combined antiinvasive and antiangiogenic therapy against high-grade gliomas. Nakabayashi and colleagues made use of the MMP inhibitor MMI-166 which significantly inhibited the invasive and angiogenic activities of glioma cells *in vitro* and *in vivo*, leading to tumor growth inhibition *in vivo* [129]. Another group tested the effects of sunitinib on orthotopic models of GBM *in vitro* and *in vivo*. Sunitinib is an oral multitargeted tyrosine kinase inhibitor with both antiangiogenic and antitumor activities due to selective inhibition of various receptor tyrosine kinases. The drug exhibited potent antiangiogenic activity; however, the antiinvasive activity of sunitinib was observed only *in vitro*, since it was not effective in overcoming the invasion increase caused by its antiangiogenic activity [130].

Recently, Conley and colleagues [131] have found, through the generation of intratumoral hypoxia in human breast cancer xenografts, that the antiangiogenic agents sunitinib and bevacizumab increase the cancer cell population. Furthermore, *in vitro* studies revealed that stem or progenitor cell enrichment is primarily mediated by hypoxia, specifically by HIF1*α*. These are very interesting results, since they demonstrated that antiangiogenic agents are able to disrupt tumor vasculature, and therefore the PVN, but meanwhile, they create neohypoxic niches, which in turn can generate new GSCs [122] and reestablish the proliferative niche, pointing out the dynamics of the PVN and HN crosstalk, and even more so because they are able to interconvert (Figure 1(b)). These findings show the importance of employing converging therapeutical strategies into both niches by, for example, aiming at both VEGF and HIFs together.

Indeed, Rapisarda et al. [125] tested the hypothesis that HIF-1*α* inhibition in a hypoxic-stressed tumor microenvironment generated by the administration of antiangiogenic agents may result in a more pronounced therapeutic effect. The activity of bevacizumab, either alone or in combination with the HIF-1*α* inhibitor topotecan, was evaluated in the glioblastoma cell line U251-HRE (containing a hypoxic responsive element) xenografts. The luciferase expression in U251-HRE xenografts is dependent on the presence of a functional HRE sequence. The authors then designed the experiments to test whether topotecan inhibited HIF-1-dependent luciferase expression and tumor growth in U251-HRE xenografts. The combination of a low dose of topotecan with bevacizumab synergically inhibited tumor growth. The addition of topotecan to bevacizumab was also associated with significant inhibition of proliferation and with induction of apoptosis (not seen with bevacizumab alone). Importantly, they showed that the increased cytotoxic activity by bevacizumab did not account for the increased antitumor effects observed. The effects of the combination of the two drugs are explained by the inhibition of the hypoxic responses usually triggered by bevacizumab. Interestingly, there was also a reduction in angiogenesis relative to either agent alone, possibly as a result of these two agents inhibiting converging angiogenic pathways controlled by HIF-1 transcriptional activity, such as the VEGF pathway.

On the other hand, it is intriguing that, although HIF-1*α* inhibition alone does not significantly affect GSC maintenance [122, 124], it still can directly modulate the GSC niche, indirectly affecting the GSC population. Therefore it would be of great value to evaluate effects of both VEGF and HIF-1*α* inhibition on GSC population. Furthermore, the effects of VEGF and HIF-2*α* inhibition on tumor growth and aggressiveness remain to be explored, since until now HIF-2*α* has been considered the main regulator of GSC in the HN [124].

Overall, there has been a significant progress in studies regarding GSC niche. The hypoxic environment was shown to regulate many aspects of GSC signaling, but little is known about they behave *in vivo* in such niches. The complex mechanisms involved in hypoxic responses and in antiangiogenic therapy, and its consequence specially in GSC maintenance must be further examined to better explore antiglioma therapy.

## 6. Part V: Epigenetic Control at the Niche

Epigenetics are referred to as the mechanisms by which gene expression is regulated without altering the genomic sequence. Epigenetic regulation can thus shape cell fate allowing adjustment to varying environmental conditions (reviewed in [131]). These molecular signals act on chromatin of not only one cell, but in the whole microenvironment [132], promoting cell-type-specific changes through the acquisition of distinct programs for gene expression. This process renders this mechanism of great importance to the developing tissue stability and homeostasis, which are accomplished by the maintenance of cellular memory (the heritable patterns of gene expression), through genomic imprinting.

Chromatin contains several proteins that are required for its assembly and packaging into euchromatin or heterochromatin, as well as for DNA replication and transcription, DNA and histone modification, and DNA repair or recombination (reviewed in [133]). The main epigenetic mechanisms include DNA methylation, histone modifications (acetylation and methylation), and regulatory noncoding RNAs (reviewed in [134]).

### 6.1. Epigenetic Mechanisms

Recent studies have highlighted the active role of histone modifications in gene expression regulation (reviewed in [135]). The covalent posttranscriptional changes at their amino-terminal tails by acetylation, phosphorylation, methylation, and ubiquitylation dictate how much access transcriptional regulators have to the DNA (reviewed in [133]). Lysine acetylation promotes nucleosome relaxation by decreasing the interaction of positively charged histone tails with the negatively charged DNA phosphate backbone. Histone deacetylases (HDACs) have an opposite activity: by deacetylating histone tails, the DNA is packed into condensed chromatin (nucleosomes) which, as a result, represses gene transcription (reviewed in [134]).

Many epigenetic studies focused on embryonic stem cell (ESC) maintenance and differentiation, relating it to embryonic development. Specific epigenetic marking by histone modifications is already known to occur in multipotent stem cells because of the binding of transcription factors involved in lineage choice (reviewed in [136]). Transcription factors that are expressed in ESCs (including Oct-4, Nanog, and Sox-2) would have a similar role in establishing epigenetic marks.

Concerning neuronal differentiation, Li and colleagues [134] have summarizedthe epigenetic influence on neuron-specific gene expression. They highlight that the recruitment of HDACs to neuronal gene promoters is essential for the repression of the same genes in nonneuronal cells and that the maintenance of histone acetylation is important for neuronal differentiation. Epigenetic mechanisms control lineage-specific gene expression for the generation of different neural cell types. Mechanisms such as DNA methylation keep GFAP repressed in neurons, but this can also be reverted in response to microenvironment changes. Furthermore, multipotent neural progenitor cells differentiate predominantly into neurons in the presence of the HDAC inhibitor (HDACi) valproic acid (VPA), and the silencing of some neuronal-specific genes can be reverted by treatment of the HDACi trichostatin A (TSA) [134].

**Figure 3:**
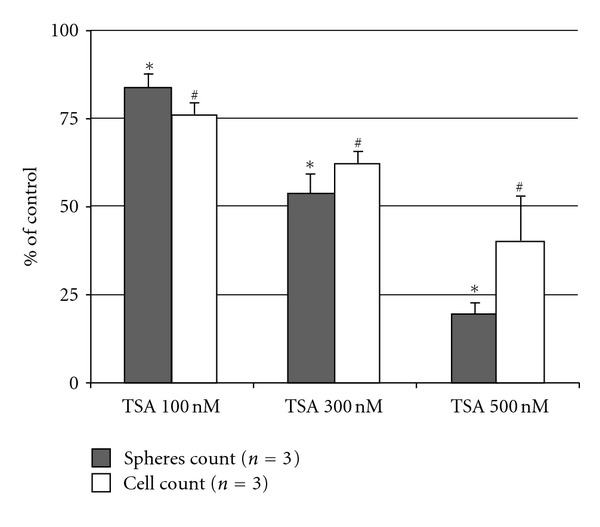
Tumorsphere formation assay upon treatment of U87-MG cells with Trichostatin A (TSA). A seventy-two-hour treatment with the histone deacetylase inhibitor TSA affects tumorsphere formation and cellular proliferation after medium shift to NSC medium. Bar represents control percentage. *represents *P* < 0.02 for 100 nM and *P* < 0.001 for 300 and 500 nM for spheres count, and ^#^represents *P* < 0.046 for 100 nM *P* < 0.011 for 300 nM and *P* < 0.001 for 500 nM for cell count as measured by trypan blue. One-way ANOVA, followed by Turkey's post hoc test were used for statistical analysis, where *P*-values <0.05 were considered significant.

Results from our laboratory show that this action may be effective against GSC propagation. Treatment for 72 hours with TSA was sufficient to decrease tumorsphere formation after medium shift to NSC medium in the human glioma cell line U87-MG, as measured by the tumorsphere formation assay (Figure 3). This result shows that acetylation may be essential for GSC stemness and maintenance.

### 6.2. Epigenetics in Tumors

Since chromatin structure responds to environmental cues and it is tightly regulated in several ways at the molecular level, tumors clearly originate from not only genetic alterations, but also from epigenetic aberrations in its microenvironment. Indeed epigenetics regulate many aspects of tumor behavior, including initiation, proliferation, and metastasis of the primary tumor [137].

As fully reviewed by Dey [138], cancer cells present aberrations in their DNA methylation pattern. Hypomethylation at centromeric repeat sequences has been linked to genomic instability. Furthermore, hypomethylation has also been associated with the activation of genes required for invasion and metastasis. On the other hand, local hypermethylation of individual genes has been associated with aberrant gene silencing, such as the repression of tumor suppressor genes. Beside that, evidences show aberrant loss or gain of histone methyltransferase (HMTase) activity in tumorigenesis and proliferation of cancer cells [138]. Moreover, histone acetylation/deacetylation in promoter regions contributes to the disregulation of gene expression and has also been associated with carcinogenesis and cancer progression [138].

In addition, DNA methylation patterns are useful as biomarkers for glioblastoma. The most relevant mark is the methylation status of the MGMT (O6-methylguanine–DNA methyltransferase, a DNA repair protein) gene promoter. When the MGMT promoter region is epigenetically silenced, it is associated with a favorable outcome after temozolomide chemotherapy in patients with newly diagnosed glioblastoma, suggesting that it could be further studied as a biomarker with prognostic value [139].

Hegi and colleagues [140] have also associated epigenetic marks to glioma cells: in their work, the authors analyzed the methylation status of both DNA cytosine methyltransferases (DNMTs) and specific tumor suppressor genes promoters and compared them with normal brain samples in order to confirm that tumor suppressor genes are hypermethylated and silenced in gliomas [141]. With their results, the authors propose that overexpression of DNMT1 and DNMT3B in gliomas is a result of a significant hypomethylation occurring in the euchromatin region of its gene promoters. The increase of DNMT activity, in turn, causes hypermethylation of various tumor suppressor gene promoters, leading to the epigenetic inactivation of those genes, enhancing the proliferative capacity of glioma cells and harboring a poor prognosis in gliomas. The authors propose that overexpression of DNMTs may serve as a marker for cancer cells and as a potential target for future cancer therapy [140]. This work is an example of how epigenetic aberrations cause genomic instability which contribute to the achievements of the tumorigenic hallmarks in gliomas, illustrated in Figure 1.

### 6.3. Epigenetic Plasticity

Epigenetic plasticity is often illustrated by the normal stem cell lineage commitment. Likewise, differentiated cells are also able to be epigenetically reprogrammed into a stem-like chromatin state, as seen in iPSCs (induced pluripotent stem cells) reprogramming, and to transdifferentiate into a disparate lineage (such as when glioma cells generate endothelial cells). The dedifferentiation of cancer cells into CSCs has also been described as epigenetic plasticity. Furthermore, the HDACi VPA has been shown to facilitate the induction of pluripotency by chromatin remodeling [142] (reviewed in [143]). VPA was also shown to be involved in neuronal differentiation of NSC, regulating neurogenesis [144].

Therefore, the ability of cells to alter their state by modulating gene expression has also been observed in differentiation-altering, microenvironment-associated plasticity [143]. This means that gene expression of cancer cells can be altered, as well as its phenotype, by alternating its microenvironment. To illustrate, transition from 2D to 3D culture reduced epigenetic plasticity in platinum-resistant CP70 ovarian cancer cells [143]. Furthermore, the influence of the tumor microenvironment components over the maintenance of the cancer cells is reinforced when tumor cells are placed in a nonmalignant environment. Melanoma cells, when plated on top of ESC-derived extracellular matrices, remarkably differentiate into sphere-forming melanocytes, and the opposite (ESC plated on top of melanoma-derived extracellular matrices) is also true [145, 146]. Human ESCs show the ability to suppress the tumorigenic phenotype by the secretion of Lefty (which is exclusively expressed in ESCs), which neutralizes the expression of Notch in aggressive tumor cells.

It still remains unclear whether abnormal epigenetic regulation is a cause or consequence of cancer. Evidences demonstrate that the environment itself can modulate epigenetic plasticity, so abnormal signals from the microenvironment could predict and sensitize a potential cell for oncogenic transformation. Other results show that cancer cells or CSCs maintain the epigenetic signature of normal stem cells, which could favor malignant transformation. On the other hand, epigenetic disregulation is often a consequence of chromatin regulatory protein abnormalities, such as histones and HDACs, which are encoded by the very same DNA sequences that they regulate. Therefore, these alterations could arise as result of the genetic instability related to cancer.

Either way, epigenetic regulation of cancer cell gene expression offers us the opportunity to modulate these responses, since these are very dynamic changes, as opposed to the permanent genetic mutations, which therefore require complex therapeutic approaches, such as gene therapy and enzymatic reposition. GBM, specially, shows remarkable plasticity, and may be susceptible to epigenetic modulators such as HDACi, which are able to diminish the tumorigenic potential of cancer cells [147–149], all the while offering new insights into how glioma cells respond to treatment. In addition, epigenetics can modulate the PVN and the HN. Hypoxic microenvironments may influence local epigenetic alterations, leading to inappropriate silencing and reawakening of genes involved in cancer, the main mechanism being loss of global methylation [150]. Potential cellular factors that link HDACis to the repression of HIF function have been proposed: type I/II HDAC inhibitors repress HIF function by either reducing functional HIF-1*α* levels or repressing HIF-*α* transactivation [149]. TSA, for example, is among several HDACi reported to repress angiogenesis *in vitro* and *in vivo* [151, 152]. VEGF is also epigenetically regulated [153], and together with the inhibition of HIF response, scientists can aim for the modulation of the GSC microenvironment to develop new therapeutic strategies.

## 7. Final Remarks

The knowledge about how neurogenesis functions in physiological conditions and maintains neuronal plasticity (which allows for physiological adaptations) lies on understanding the peculiarities of the mitotic niches that allow for stem and progenitor cells to proliferate and generate new cells. Depicting the function of normal stem cells and their relationship with their surroundings (a crucial crosstalk for tissue homeostasis) facilitates the understanding of cancer stem cell functions. Hence, it can awake new insights into cancer therapy, because accumulating evidences point out to CSCs as the main culprit. It is clear that both physiological and pathological stem cell niches share similar features, such as hypoxic and angiogenic signaling, as well as several other pathways which enable cancer cells to proliferate and self-renew with no limitations.

Through the study of neurogenesis, researchers could also shed light into the origins of glioblastoma. Such incurable malignancies are very heterogeneous and dynamic, hampering the complete elucidation of tumor biology during the first stages of their inception. The characterization of neural progenitors in specific brain niches lead to studies which focused on specific cell types. Through the advent of modern techniques, it was also possible to trace markers and cells along a certain period. As mentioned above, the cell of origin for GSCs is still under debate, but it is now becoming clear that they may arise from OPCs and NSCs from the neurogenic niches. Likewise, they may arise from mature cells that acquired the ability to self-renew as a result of oncogenic mutations; it is important to point out that this still remains an open question.

The way by which the microenvironment affects its cells and vice versa is still being uncovered, but the deeper the scientists unravel the idiosyncrasies of epigenetic regulation, the more is understood about how a cell responds to each context. This notion is already raising new promising pharmacological approaches for cancer therapy, since reverting epigenetic aberrations possibly inhibit the cancer-prone state (Figure 1(b)). Modulators such as histone deacetylases inhibitors, which are already being employed in clinical trials for several malignancies, are capable of differentiating CSCs, diminishing their malignant potential. Furthermore, new discoveries regarding the inhibition of angiogenic factors, such as VEGF, and the blockade of signals which arise from the hypoxic niche are also promising for targeting CSC niches. Even though much work still needs to be accomplished in order for researchers to uncover the dynamics of tumor microenvironments with its cells, this area has provided important information regarding tumor behavior, and new therapeutic approaches can now focus not only on the tumor itself, but also on its surrounding tissue.
